# Perioperative Treatment in Resectable Gastric Cancer: Current Perspectives and Future Directions

**DOI:** 10.3390/cancers11030399

**Published:** 2019-03-21

**Authors:** Angelica Petrillo, Luca Pompella, Giuseppe Tirino, Annalisa Pappalardo, Maria Maddalena Laterza, Marianna Caterino, Michele Orditura, Fortunato Ciardiello, Eva Lieto, Gennaro Galizia, Carlo Castoro, Ferdinando De Vita

**Affiliations:** 1 Division of Medical Oncology, Department of Precision Medicine, School of Medicine, University of study of Campania “Luigi Vanvitelli”, Via Pansini n.5, 80131 Naples, Italy; luc32@hotmail.it (L.P.); giuseppett@icloud.com (G.T.); annalisa.pappalardo88@gmail.com (A.P.); marilena_laterza@yahoo.it (M.M.L.); caterinomarianna@gmail.com (M.C.); michele.orditura@unicampania.it (M.O.); fortunato.ciardiello@unicampania.it (F.C.); 2 Division of GI Tract Surgical Oncology, Department of Surgical Sciences, University of study of Campania “Luigi Vanvitelli”, Via Pansini n.5, 80131 Naples, Italy; eva.lieto@unicampania.it (E.L.); gennaro.galizia@unicampania.it (G.G.); 3 Department of upper GI surgery, Humanitas Research Hospital-Humanitas University, 20089 Rozzano (Milano), Italy; carlo.castoro@hunimed.eu

**Keywords:** gastric cancer, perioperative treatment, neoadjuvant chemotherapy

## Abstract

Gastric cancer (GC) is the fifth-most common cancer worldwide and an important cause of cancer-related-death. The growing knowledge of its molecular pathogenesis has shown that GC is not a single entity, but a constellation of different diseases, each with its own molecular and clinical characteristics. Currently, surgery represents the only curative approach for localized GC, but only 20% of patients (pts) showed resectable disease at diagnosis and, even in case of curative resection, the prognosis remains poor due to the high rate of disease relapse. In this context, multimodal perioperative approaches were developed in western and eastern countries in order to decrease relapse rates and improve survival. However, there is little consensus about the optimal treatment for non-metastatic GC. In this review, we summarize the current status and future developments of perioperative chemotherapy in resectable GC, attempting to find clear answers to the real problems in clinical practice.

## 1. Introduction

Gastric cancer (GC) represents the fifth-most common tumor and the third-leading cause of cancer-related death worldwide [1], showing similar trends in Europe [2]. Even if GC was considered a single entity in the past, today it is well-known as a constellation of distinct diseases that can be classified from different points of view, as comprehensively reviewed by Tirino et al. [3]. In fact, moving from the first histological GC classification provided by Lauren et al. [4], The Cancer Genome Atlas (TCGA) work [5] has definitively clarified that the clinical and prognostic differences observed between the intestinal and diffuse GC types have very peculiar molecular bases, with specific clinical implications.

Surgery with a D2 lymphoadenectomy, removing at least 25 lymph nodes [6] and without macro- or microscopial residual (so-called R0 resection), represents the only curative approach for resectable GC. However, in western countries the mortality remains very high due to the lack of specific screening programs and more than 50% of GC are diagnosed as locally advanced disease, which are not suitable for an upfront curative surgical approach. Moreover, even in case of curative resection, the prognosis of patients (pts) with a node positive status at diagnosis remains poor, with five-year survival rates of 20–30%. In this context, perioperative and adjuvant approaches have been developed over the last decades as part of a multimodality treatment in order to decrease local and distant relapses after gastrectomy and improve survival rates. However, little consensus exists on the optimal strategy for resectable GC.

In this review, we summarize the current status and future developments of perioperative multimodality treatment for resectable GC, reporting the “state of the art” about the role of radiotherapy, early metabolic assessment, molecular prognostic and predictive factors and new biological or immunological agents, and trying to offer clear answers to the real problems in clinical practice.

## 2. Perioperative Chemotherapy in Resectable GC: The “State of Art”

The need to find a way to improve survival in western GC pts has led to the creation of the so-called “perioperative” strategy. The aim of this approach is to improve overall survival (OS) by downstaging the tumor, improving pathological responses and reducing the risk of local and distant relapses, thus eradicating the micrometastatic disease.

The landmark trials in the setting of perioperative chemotherapy for resectable GC are MAGIC [7] and FNCLCC/FFCD ACCORD [8]. In the MAGIC Trial [7], ECF (epirubicin, cisplatin and 5-fluorouracile) was tested as a perioperative treatment (three pre- and three postoperative cycles) compared to surgery alone in 503 pts with resectable adenocarcinoma of the stomach (74%), esophagogastric junction (11–12%) and lower esophagus (14–15%). The results were in favor of experimental arm with hazard ratio (HR) for death of 0.75 (95% Confidence interval (CI) 0.60–0.93, *p*: 0.0009) and five-year survival rate of 36% and 23% in perioperative and control arm, respectively.

On the other hand, the FNCLCC/FFCD ACCORD trial [8] evaluated the role of perioperative treatment with cisplatin plus 5-fluorouracil (CF: two or three preoperative cycles and three or four postoperative cycles) compared to surgery alone in 224 pts affected by resectable adenocarcinoma of the stomach (25%), esophagogastric junction (GEJ) (64%) and lower esophagus (11%). Once again, the results were in favor of experimental arm, with an HR for death of 0.69 (95% CI, 0.50–0.89, *p*: 0.02) and five-year survival rate of 38% (perioperative arm) versus 24% (control arm). The trial showed similar positive results for disease free survival (DFS), with HR for disease recurrence of 0.65 (95% CI, 0.48–0.89, *p*: 0.003). Both were multicenter trials involving different centers in the same nation (England for MAGIC and France for FNCLCC/FFCD ACCORD) over a period of 10 years (1995–2005), but randomizing a different kind of pts according to primary tumor location (more cardial cancer in French trial and more GC in the English study). However, both MAGIC and ACCORD demonstrated a significant improvement in OS in pts treated with perioperative chemotherapy versus surgery alone, regardless of tumor locations, making this strategy a standard of care in Europe, at least.

A reasonable clinical doubt, considering the similar positive results of MAGIC (anthracycline based regimen) and FNCLCC/FFCD ACCORD (not anthracycline based regimen), is to add or not anthracycline in peri-operative setting. In this context, the MRC OE05 Trial [9] showed that the intensification of treatment with four preoperative cycles of ECX (platinum, capecitabin and epirubicin) does not increase survival in pts with surgically resectable oesophageal adenocarcinoma compared to two preoperative cycles of CF, making the anthracycline-free regimen the treatment of choice. However, it is important to underline that the OE05 trial enrolled only pts with proximal adenocarcinomas (oesophageal adenocarcinoma, Siewert I and II cancers), excluding the distal ones.

The most recent clinical trial conducted in the field of perioperative approach is the German FLOT-4 study, whose results were presented as oral presentations at ASCO 2017 [10] and ESMO 2017 [11] meetings, but not published in extenso yet. In this multicenter and mono-national trial, the MAGIC regimen was compared with a taxan-containing triplet (FLOT schedule: 5-Fluorouracil, Oxaliplatin and Docetaxel) in 716 pts affected by gastric (44%) or junctional (Siewert I-II-III, 56%) non-metastatic adenocarcinoma. FLOT was administered for four pre and four postoperative cycles, showing an impressive improvement in median progression free survival (PFS) compared to ECF (30 versus 18 months, HR: 0.75; 95% CI: 0.62–0.91, *p*: 0.004) and median OS (50 versus 35 months, HR: 0.77; 95% CI: 0.63–0.94, *p*: 0.012). Moreover, projected five-year OS rates were 45% in experimental arm and 36% for ECF/ECX, in strong accordance with the previous data presented in MAGIC study [7]. The benefit of FLOT was shown in all the subgroups analyzed, such as proximal versus distal tumors, well versus poorly differentiated as well as in the early stages (cT1,2), in which FLOT showed greater survival benefit than ECF/X. Of note, in case of tumors with signet ring cells component (that are known to be poorly responsive to chemotherapeutic agents), FLOT showed a better outcome than standard ECF/X, supporting the use of this regimen for poorly cohesive tumors. Finally, there was no increase in surgical morbidity and mortality in FLOT arm (55% in both arms). Based on these results, the FLOT regimen could be considered the new standard chemotherapy regimen for perioperative strategy of resectable GC, with clear demonstration that, in this setting, anthracyclines should be definitively abandoned (Table 1). However, the publication of the final analysis in extenso is awaited in order to include this regimen in the recommendations of guidelines.

Therefore, international clinical practice guidelines [12,13] suggest that GC pts with clinical T1, N0 tumors should proceed directly to surgery, administering adjuvant therapy if the pathological stage requires it, whereas pts with T3-4 or N+ tumors (the majority of western pts) should be treated within a perioperative strategy, which today includes anthracycline-free regimens, such as the FLOT schedule. The choice of appropriate treatment in case of T2 is still under debate, even if the most recent findings suggest the use of a perioperative approach. However, it is important to underline that all pts should be treated in dedicated centers with high volume and high expertise in the management of GC in order to decrease surgical morbidity and mortality and improve postoperative care for these pts.

## 3. The Role of Adjuvant Phase of a Perioperative Approach

**Figure 1:**
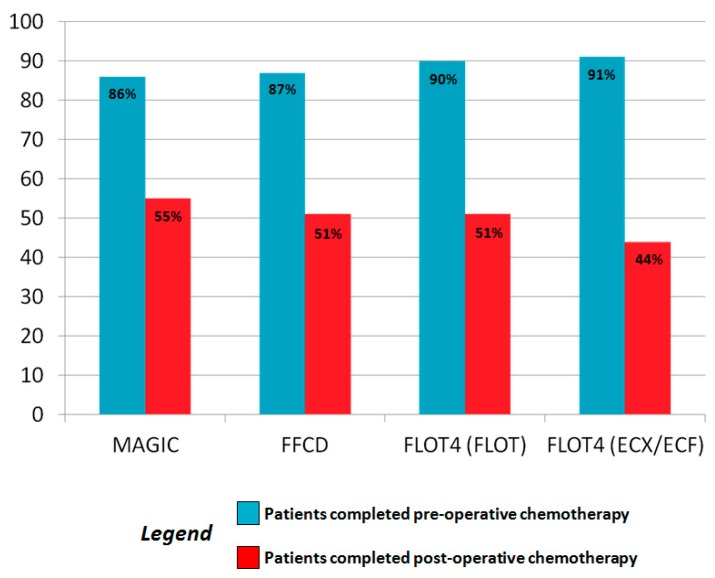
Rates of patients that received pre and postoperative treatment in the principle Phase III of MAGIC [7], FFCD-ACCORD [8], and FLOT-4 [10,11] clinical trials.

A very intriguing and open question in the field of perioperative treatment is how important is the adjuvant part of this approach. In fact, only almost 50% of pts randomized in the MAGIC [7] and FNCLCC-ACCORD [8] trials completed the postoperative treatment, as well as in the FLOT-4 trial [10,11] (44% and 51% in the standard and experimental arm, respectively) (Figure 1). In all these studies, the authors explained that these rates were reported due to early death of pts after surgery, disease progression, postoperative complications, or previous toxic effects of the preoperative part of the treatment.

In the MAGIC study [7], the authors concluded that is not possible to attribute the favorable outcome of the experimental arm to either preoperative or postoperative chemotherapy or both. Moreover, randomized trials evaluating the role of upfront neoadjuvant chemotherapy plus surgery versus a complete perioperative approach have been not published yet and only retrospective experiences are available in literature today. Mirza et al. [14] conducted the first attempt to resolve this issue, selecting 66 GC pts that received chemotherapy according to MAGIC [7] protocol, followed by surgery. Only 47% (31 pts) underwent the adjuvant part of the therapy, while 53% (35 pts) did not receive any treatment due to postoperative complications, refusal, or large time elapsed between surgery and the beginning of chemotherapy. The results showed a significant increase in survival for pts treated with both pre and postoperative chemotherapy, although with a very small sample size, which represents the main limitation of this analysis.

A larger observational study about this topic was conducted on 134 consecutive German pts [15], which confirmed the superiority of both pre- and post-surgery chemotherapy in terms of outcomes. It analyzed the role of perioperative approach using FLOT, EOX, ECX versus the preoperative phase only and provided evidence that the administration of postoperative chemotherapy could contribute to survival benefit in a statistically significant way (five-year survival was 75.8% versus 40.3% in pts who received both phases or only the preoperative part of treatment, respectively). Noteworthy, adjuvant treatment was shown to be an independent prognostic factor related to survival at multivariate analysis, observing the largest benefit in pts with node-positive disease or sub-optimal histological tumor regression grade (TRG). Therefore, these results suggest that the subgroup of pts with unfavorable prognostic factors after surgery could benefit greater from a complete perioperative approach. However, these considerations should be confirmed with prospective randomized clinical trials.

Positive results in favor to a complete perioperative treatment were also shown by a meta-analysis reporting the data from 2093 GC pts randomized in 14 clinical trials [16]. The global analysis showed a statistically significant benefit in OS for the combination arm compared to adjuvant treatment alone (HR: 0.48, 95% CI: 0.35–0.67; *p* < 0.001) as well as in PFS and rate of R0 resections.

On the other hand, a recent retrospective German analysis [17] provided an opposite opinion about a complete perioperative treatment. The authors selected 299 pts who underwent neoadjuvant chemotherapy followed by surgery, of which 56.8% (170 pts) received the adjuvant part of this approach. The completion of the entire perioperative approach did not improve significantly OS (78.2 months versus not reached (NR), *p*: 0.331) and recurrence-free survival (RFS: 43.3 versus 41.1 months, *p*: 0.118), but the adjuvant part seemed to increase RFS only in pts treated with FLOT regimen (*p*: 0.038) or with non-intestinal tumors (*p*: 0.023), supporting the need for further investigations.

In this context, the NeoFLOT phase II trial [18] investigated the role of FLOT regimen as pure neoadjuvant treatment (6 preoperatory cycles). The trial showed a very high rate of pathologic complete response (pCR: 20%) and R0 resection rate (86%), in line with the results previous reported in the MAGIC [7] and FNCLCC-ACCORD [8] trials.

In conclusion, the role of the postoperative part of perioperative approach is not clear, although many studies suggest that a complete perioperative course could improve survival. Future randomized phase III trials, such as NAGISA [19] (UMIN000024065) and PRODIGY [20] (NCT01515748) could clarify this issue.

## 4. The Role of Radiation Therapy within Perioperative Approach

In the context of GEJ cancer, the comparison between preoperative chemo-radiotherapy and chemotherapy alone is an open challenge and the choice between these two strategies depends mainly on physician preference. No clear evidence and no direct comparison is currently available and most assumptions are obtained indirectly from subgroup analysis included in the main clinical trials. The majority of the studies, in fact, has been always designed to compare a defined neoadjuvant approach (chemotherapy or chemo-radiotherapy) with surgical resection alone.Based on these evidences, international guidelines [12,13] recommend preoperative chemo-radiotherapy and chemotherapy as equivalent alternatives for the preoperative treatment of locally advanced GEJ adenocarcinomas.

One of the few example of study in this field is the POET trial [21], in which 126 pts with locally advanced (T3–T4) GEJ adenocarcinoma (Siewert I-II-III) were randomized to receive an induction chemotherapy or chemotherapy followed by chemo-radiotherapy before surgery resection. Unfortunately, the study was closed prematurely due to slow accrual. The addition of radiation therapy improved the three-year survival rate from 27.7% to 47.4% (HR: 0.67) and the amount of pts undergoing complete tumor resection was not different in the treatment groups, showing a higher percentage of pCR in the radiotherapy arm (15.6% versus 2.0%). Although statistical significance was not achieved (*p*: 0.07), this study showed some benefits when including radiotherapy into preoperative treatment, and it paved the way for further investigation. Long-term follow-up data [22] confirmed a trend in favor of preoperative chemo-radiotherapy at five years (39% versus 24.4%; HR: 0.65; *p*: 0.055), despite of the lack of statistical significance but with a significant improvement in local PFS with radiotherapy (HR: 0.37; *p*: 0.01).

As anticipated, given the lack of solid data, relevant indications can be achieved by the analysis and comparison of large trials currently available in this field, reading the results with caution in the light of indirect comparison of very different trials. MAGIC trial [7], FNCLCC-ACCORD [8], EORTC 40954 [23], CALGB 9781 [24] and CROSS trial [25,26] could be considered the largest reference studies that should be compared. It is important to underline the results of the CROSS trial [25,26], which showed that the addition of weekly carboplatin (area under the curve of 2 mg/mL/min) and paclitaxel to concurrent RT (41.4 Gy in 23 fractions, 5 days per week) as neoadjuvant treatment improves survival (median OS: 49.4 versus 24.0 months in the neoadjuvant and in the surgery group, respectively; HR: 0.657; 95% CI: 0.495–0.871; *p*: 0.003) among pts with resectable esophageal or GEJ tumors (cT1N1 or T2-3N0-1). These trials have been summarized in Table 2. 

The overall population included in these trials reached almost 1300 pts and included different tumor sites: MAGIC trial [7] mostly included GC (75%); the majority of FNCLCC-ACCORD [8] pts had a GEJ cancer; CALGB [24] and CROSS [25,26] were mainly based on esophageal cancer and EORTC [23] included Siewert II/III adenocarcinoma (53% of cases). The evaluation of tumor sites seems to be crucial to analyze the results and to understand the findings of these trials.

Based on the survival data reported in Table 2, it appears reasonable to consider that the outcome between different approaches (chemotherapy or chemo-radiotherapy) is similar. In fact, the survival benefit related to perioperative/preoperative chemotherapy alone or chemo-radiotherapy may be considered equivalent and the addition of radiation therapy does not seem to offer an improved survival outcome. However, if we consider the presence of squamous cell carcinoma of the esophagus in these trials, we can clearly see a major benefit of radiation therapy for esophageal squamous cells carcinoma, when compared to adenocarcinoma (HR: 0.48 and 0.73 in squamous cell and adenocarcinoma, respectively [25,26]). Therefore, in the choice of treatment (perioperative chemotherapy or neoadjuvant including radiotherapy), the clinical and biological differences related to different tumor location and subtype should be taken into account.

Another important issue regarding the radiation therapy within a perioperative approach is the role of radiotherapy in the postoperative part of multimodality treatment. The CRITICS trial [27] randomized 788 radically resected stage IB-IVa GC pts already treated with three cycles of neoadjuvant chemotherapy (EOX/ECX) to receive the same postoperative chemotherapy (three cycles) or postoperative chemo-radiotherapy (45 Gy concomitant with weekly cisplatin and daily capecitabine). There was no difference in five-year OS rates (42% versus 40%, *p*: 0.90), proving that a more intensive postoperative treatment does not improve outcome. To note, in MAGIC [7], FNCLCC-ACCORD [8] and FLOT-4 [10,11] trials, only about 50% of the pts completed the planned adjuvant therapy.

In the light of these findings, the final results from ongoing trials are expected, such as the phase III TOPGEAR [28] and ESOPEC [29], which seek to compare, respectively, perioperative ECF treatment with or without preoperative chemo-radiation for resectable GC and perioperative FLOT or chemo-radiotherapy according to CROSS schedule [25,26] for esophageal or GEJ adenocarcinoma.

## 5. The Role of Target and Immunotherapy in the Perioperative Setting

The research focused on the use of target agents in the neoadjuvant approach in order to improve the outcomes for these pts [3,30], based on the results reported in the metastatic setting (Table 3).

Moving from the landmark ToGA trial [31], an exploratory analysis of MAGIC showed that Her-2 status was neither a prognostic nor predictive factor in the study population (HR: 0.74 and 0.58 for Her-2 positive and negative, respectively; *p*: 0.7), concluding that, unlike the metastatic disease, Her-2 did not represent an independent prognostic biomarker for early stages [32].

The Phase II NEOHX study [33] and the HER-FLOT trial [34] investigated the addition of trastuzumab for perioperative treatment with XELOX (capecitabin and oxaliplatin) and FLOT schedule, respectively, for Her-2 positive gastric or GEJ cancer. Both these trials showed a promising safety profile and activity for the schedules, reporting 8.3% and 22.2% of pCR in the NEOHX and Her-FLOT trial, respectively. The PETRARCA (NCT02581462) and INNOVATION (NCT02205047) trials are currently investigating the role of the addition of trastuzumab and pertuzumab to FLOT (PETRARCA) and trastuzumab or trastuzumab plus pertuzumab to cisplatin plus capecitabine (INNOVATION) in the perioperative setting.

Regarding the antiangiogenic biological agents, the phase II/III UK MRC ST03 trial investigated the addition of bevacizumab to ECX as perioperative treatment for pts with GEJ adenocarcinoma, showing no benefit in OS at three years in the combination arm (HR: 1.08, 95% CI 0.91–1.29; *p*: 0.36) [35]. Moreover, bevacizumab was associated with impaired wound healing and leak of the anastomosis after surgery (12% versus 7% in control arm), concluding that the addition of this biological anti-vascular endothelial growth factor to chemotherapy is not recommended in the perioperative setting, as well as previously reported in AVAGAST trial for metastatic disease [36]. Another antiangiogenic agent, ramucirumab, was shown to improve OS and PFS in second line treatment for pts with metastatic GC, as reported in the REGARD [37] and RAINBOW trials [38]. Based on these findings, the RAMSES trial (NCT02661971) is currently investigating the role of FLOT with or without ramucirumab in the perioperative treatment of Her-2 negative gastric and GEJ adenocarcinomas.

Finally, research is focusing on the role of immunotherapy for GC in the perioperative setting. In this light, the Phase III KEYNOTE-585 study (NCT03221426) seeks to evaluate the efficacy and safety of pembrolizumab plus chemotherapy compared with placebo plus chemotherapy (according to CF or FLOT schedule) as a perioperative treatment for localized gastric or GEJ adenocarcinoma. Moreover, the Phase I/II ICONIC trial (NCT03399071), which seeks to investigate the safety and efficacy of avelumab in combination with FLOT, is ongoing.

In conclusion, all the biological agents investigated in current international trials have failed to improve the outcomes for these pts when administered in addition to standard treatment. Moreover, a series of trials evaluating the role of biological and immunological agents in this field are ongoing. For these reasons, the use of target or immunotherapy in the perioperative setting is exclusively experimental and does not represent the standard of care today. However, results of the ongoing trials are awaited to clarify this issue.

## 6. Particular Sections

### 6.1. The Impact of Histology in the Choice of Treatment

GC is a heterogeneous disease as reported in the classifications that have been developed over the years [3]. Lauren’s classification firstly reported the distinction between diffuse (with or without signet ring cells component) and intestinal types, with different epidemiologic, pathogenetic, biological features and clinical behavior [4]. However, today there is no difference in the clinical management of these two histotypes, although the intestinal type seems to have a better prognosis [39]. A recent meta-analysis, comprising the data from 73 published studies on 61.000 pts, confirmed that GC with diffuse-type histology had a worse prognosis than those with intestinal-type (HR: 1.23; 95% IC: 1.17–1.29; *p* < 0.0001) in loco-regional and advanced stages (HR:1.21; 95% IC: 1.12–1.30, *p* < 0.0001 and HR: 1.25; 95% IC: 1.046–1.50; *p*: 0.014, respectively), with or without (neo)adjuvant treatment [40].

Regarding the perioperative field, the first retrospective analysis, which was conducted by a French group, showed interesting results after evaluation of 3010 pts affected by GC between 1997 and 2010 [41]. Among the 924 pts with signet ring cell adenocarcinoma (30%) and treated with a curative intent, 171 pts received perioperative chemotherapy (CF doublet or triplet regimen) followed by surgery, whereas 753 pts underwent direct surgery. The median survival was statistically significant shorter in the perioperative group (12.8 versus 14 months, *p*: 0.043) and the perioperative approach was shown to be an independent predictor of poor survival. Another German analysis [42] confirmed that signet ring cell histology was significantly associated with lower probability of R0 resection and worse histopathological response (16.3 versus 28.9 %, *p* < 0.001) in pts affected by resectable gastric and GEJ cancer and treated with preoperative chemotherapy (CF-based, with taxans or epirubicin). It is noteworthy that the prognosis of these pts was significantly worse in comparison with other adenocarcinomas, making the presence of signet ring cell an independent prognostic factor.

Recently, in the phase II of FLOT4 trial [43], triplet-based schedules were shown to improve the overall pathological response—reported as TRG 1a—in intestinal histotype (23% and 10% in FLOT and ECF/X arms, respectively) versus 3% in both groups in the case of diffuse one. Moreover, in the Phase III results [10,11] the subgroup analysis showed that FLOT regimen was effective also in case of signet ring cells (HR: 0.74 versus 0.79 in the intestinal subgroup, *p*: 0.0037), encouraging the use of this regimen for poorly cohesive tumors.

Therefore, based on the available literature data [40,44], the exclusion of diffuse GC with or without signet ring cells from neoadjuvant chemotherapy is not justified. In fact, despite the lack of validated indications about the correct approach to use for pts with GC according to histotype, chemotherapy seems to be effective also in case of diffuse GC with signet ring cells. Nevertheless, these results suggest the need for dedicated clinical trials in resectable diffuse and/or signet ring cells adenocarcinomas. In this context, the ongoing PRODIGE19 trial (NCT01717924) seeks to randomize pts with resectable tumors with signet ring cells with a perioperative approach with ECF versus an upfront surgery followed by adjuvant treatment with six cycles of the same chemotherapeutic agents [45].

### 6.2. Precision Medicine in Gastric Cancer

The correct selection of pts for surgery or a multimodality treatment represents one of the most important and debated points in the field of perioperative approach for GC today. In fact, although the landmark trials had shown that pts who respond to primary chemotherapy have a better survival than those who did not show any response [7,8,10,11], it is impossible to predict how many and which pts will be in the “better prognosis” group.

#### 6.2.1. The Role of 18-Fluorodeoxyglucose Positron Emission Tomography in Perioperative Assessment

Among the different kinds of prognostic and/or predictive factors that have been investigated over the last few years, some trials have focused on the role of 18-fluorodeoxyglucose positron emission tomography (18-FDG-PET) scan as a predictor of early response to neoadjuvant treatment. The first evidence in this field was by Lordick F. et al., who evaluated in the Phase II MUNICON trial [46] the metabolic response of 18-FDG-PET in 110 pts affected by locally advanced Siewert I and II GEJ adenocarcinoma after two weeks of CF-based induction chemotherapy. They defined the metabolic response as a decrease of 35% or more in standardized uptake value when compared with the 18-PDG-PET assessed at diagnosis and distinguished between responder and non-responder respectively candidate to continue neoadjuvant treatment or to undergo to surgery. This trial showed that the responders had a significantly improved OS, when compared to metabolic non-responders (NR versus 25.8 months, HR: 2.13, 95% CI: 1.14–3.99, *p*: 0.015), identifying 18-FDG-PET as a feasible instrument to guide the choice of a tailored multimodality approach for these pts.

Based on these results, the MUNICON 2 trial was conducted with the aim of improving the outcome of non-responder pts by using a salvage neoadjuvant radiochemotherapy after the induction period, whereas the responders were candidate to neoadjuvant chemotherapy alone before surgery [47]. Unfortunately, the primary end-point of the study—the increase of R0 resection in the responder’s group—was not met (*p*: 0.51). However, it showed an increase on histopathologic response after salvage treatment in the non-responders group, even if the prognosis of this group of pts remained poor, showing a one-year progression-free rate of 57% ± 10% compared to 74% ± 8% in PET responders (*p*: 0.035) and 2-year OS rate of 42% ± 11% compared to 71% ± 8% in the responders group (HR: 1.9; 95% CI: 0.87–4.24; *p*: 0.10). These results suggest that 18-FDG-PET could help the selection of pts with a prognostic meaning rather that predictive, since it marks the biological aggressiveness of the tumors in non-responder pts, leading to a worse outcome. Nevertheless, the use of 18-FDG-PET as predictor of response to treatment or prognosis had some limitations. First, early PET evaluation of response was not able to select pts at diagnosis, but it can distinguish responders from non-responders only after the first cycle of pre-surgical treatment. Moreover, it is known that the FDG uptake differs according to tumor histology, with a better uptake in case of intestinal type and limited value in case of the diffuse one. Therefore, the use of 18-FDG-PET as a prognostic or predictive factor needs further validations before using it in daily clinical practice.

#### 6.2.2. New Molecular Biomarkers

The genomic and molecular characterization of tumor is another way to try to improve the selection of pts at diagnosis and to guide the choice of tailored treatments. Among different classifications that have been proposed over the years [3], the most comprehensive analysis was described in the TCGA [5] and Asian Cancer Research Group (ACRG) classifications [48]. An in-depth description of these classifications is not in the aim of this review; however, although both classifications have not found applications in daily clinical practice and further studies are needed to translate these findings for the management of pts, they offer interesting “food for thought”. It is important to note that only ACRG reported different prognostic informations for each GC’s subgroup and that both the classifications identified microsatellite instability (MSI) as a distinct GC subtype.

The prognostic and predictive role of microsatellites and mismatch repair proteins (MMR) is well defined in colon cancer [49,50]. In GC, the rate of MSI ranges from 3.5% to 33.3% according to the study population or the type of molecular classification used, but its role is less clear than in colon cancer. In fact, the majority of studies in this field are retrospective and focus on the prognostic role of these factors [51,52]. In this context, the emerging key points to discuss are the role of microsatellites and MMR status as prognostic factors in localized GC, as well as their role as predictive biomarkers. Regarding the first point, Polom K. et al. revised in their meta-analysis 48 studies about MSI in pts with GC who underwent surgical treatment without neoadjuvant therapy, showing an MSI rate of 9.2% in the overall population, with a high rate in woman, age >65 years old, intestinal tumor type, middle/distal gastric location, absence of lymph node metastasis and early TNM stage (I and/or II) [53]. Moreover, pts with MSI status showed a better OS than pts with microsatellite stability (MSS) (HR: 0.69, 95% CI: 0.56–0.86; *p* < 0.001). Taking into account the good clinic pathological characteristics of these kind of tumors, the prevalence of older age and the fact that these cancers did not benefit from the use of adjuvant treatment compared to MSS ones, the determination of microsatellites status could become important at the time of diagnosis in order to select pts that could benefit from adjuvant chemotherapy. However, further studies are needed to validate these assumptions in the adjuvant setting.

Regarding the predictive role of MMR protein status, few data are available in the literature today and a recently published exploratory analysis of the MAGIC trial represents the most consistent study in this field [54]. The authors investigated the association between MMR, microsatellites status, and survival in 303 pts with resectable GEJ tumor treated with surgery upfront or perioperative treatment (ECX, according to the MAGIC design) followed by surgery. This post hoc trial analyzed both the microsatellites status by genomic DNA evaluation and the MMR protein status by immunochemistry for MLH1 (MutL homolog 1), mutS homologue 2 (MSH2), mutS homologue 6 (MSH6) and PMS1 homologue 2 (PSM2) proteins, which are the four proteins validated in colon cancer to define MMR status. However, only specimens after surgery where analyzed and, for this, the results should be read with caution in the light of a retrospective analysis. MSI high (MSI-H) status was reported in 8.5% of pts with the same clinic-pathological characteristics as previously described; the concordance between MSI-H and MMR deficiency (MMRD) was globally 97.6%. Pts with MSI-H status, and/or MMRD had a better outcome than pts with MSI low (MSI-L), MSS or MMR proficient (MMRP) when treated with surgery alone (median OS: NR in MSI-H/MMRD versus 20.5 months in MSS/MSI-L/MMRP (HR: 0.42; 95% CI: 0.15–1.15; *p*: 0.09)). Nevertheless, pts with MSI-H and/or MMRD had a worse prognosis when treated with perioperative chemotherapy followed by surgery (median OS: 9.6 months in MSI-H/MMRD versus 19.5 months in MSS/MSI-L/MMRP (HR: 2.18; 95% CI: 1.08–4.42; *p*: 0.03)). This finding is in accordance with data regarding the pathological responses after chemotherapy and surgery reported in this analysis. In fact, no pts with MSI-H and/or MMRD had a major pathological response, according to Mandard [55], whereas it was 16.3% TRG 1 or 2 in the MSS or MSI-L population and 14% in MMRP one. However, we should consider that in this post hoc study, only GC showed an MSI or MMRD status compared to the GEJ tumors (0%), which is in accordance with low prevalence of the deficiency in proximal gastric or esophageal cancers [5].

Nevertheless, these findings should be validated before representing a basis to develop a “clinical practice change” system to select pts affected by resectable GC at diagnosis. In fact, if confirmed, it could suggest that pts with MSI-H and/or MMRD may have a detrimental effect with perioperative chemotherapy and could be treated with an upfront surgical approach. On the other hand, pts with MSI-L, MSS and/or MMRP may have a better outcome when treated with chemotherapy first. The possible explications for these results might be that the MSI-H/MMRD tumors have a good prognosis, reflecting a good clinic-pathological profile; or that chemotherapy might have a negative effect on high immune infiltration in the MSI-H tumor microenvironment, destroying the immune cells that naturally acts as tumor suppressors.

Another point of increasing interest is the impact of the recently recognized Epstein Barr Virus (EBV) positive GC subgroup [5] on the selection of pts to candidate for immunotherapy with checkpoint inhibitors. It is becoming clear that EBV+ and MSI tumors are mutually exclusives entities, although both benefit greatly from the addition of Pembrolizumab, at least in advanced stages, as showed in the recently published Phase II Korean trial [56]. In particular, authors have reported an ORR of 85.7% in MSI-H and 100% in EBV-positive tumors in a cohort of heavily pretreated metastatic GC pts. This trial showed for the first time the role of these biomarkers as predictors of response to immunotherapy. However, no data exists yet in early GC setting and the role of EBV as molecular marker is still to be defined.

In conclusion, future evaluations on the determination of these biomarkers with a validated test in the specimen at diagnosis (e.g., biopsies), as well as the design of prospective randomized trial for perioperative treatment are needed to clarify the potential role of EBV, microsatellites, and/or MMR statuses as predictive and prognostic factors in GC. Actually, the determination of these biomarkers is not a part of daily clinical practice for treatment of early GC and it is recommended only in one international guideline [12] for cases of advanced stage candidates as a second- or third-line treatment with the novel immunotherapeutic agent, pembrolizumab.

#### 6.2.3. pCR as Surrogate Marker of Survival: Myth or Reality?

The availability of factors that might objectively provide information about the efficacy of perioperative treatments and the outcome of pts is an open challenge in the era of multimodality approach for GC. In this context, pathologic tumor response may represent a possible key factor. TRG systems have been established in order to assess treatment efficacy and to score clinical and prognostic outcomes, even if its interpretation and relevance remain controversial. In general, pts with complete pathological regression have a better outcome, due to the better treatment response and the ideal best disease control of micrometastatic disease.

Due to the differences between many TRG systems, in 2012 Mirza et al. [57] compared Mandard’s [55], Ninomiya’s [44] and Becker’s TRG [58,59], assessing that Mandard’s and Becker’s were the most useful in terms of survival prediction, even if less reproducible for GC [60]. These conclusions were confirmed in a recent Asiatic retrospective study on almost 200 pts affected by GC and previously treated [61].

However, the predictive role of TRG is still under debate. In fact, another retrospective analysis did not confirm the prognostic meaning of this factor in a large cohort of 800 neoadjuvantly treated GEJ adenocarcinomas [62]. Another evidence in this direction results from the analysis of Smyth et al. conducted on 330 resection specimens, according to the Mandard score, in pts treated with perioperative approach in the MAGIC trial [63]. In this study, five-year OS was significantly greater in pts with TRG1–2 than TRG 3–5 (58.8% versus 28.9%, respectively). Univariate analysis demonstrated that both TRG (1–2 versus 3–5) and lymph node status (node-negative versus node-positive) were significantly associated with OS (Mandard TRG 3, 4, or 5: HR: 1.94; 95% CI: 1.11–3.39; *p*: 0.0209; lymph node metastases: HR: 3.63; 95% CI: 1.88 to 7.0; *p* < 0.001). Multivariate analysis, including TRG and lymph node status, showed that only lymph node status was an independent predictive factor for OS (HR: 3.36; 95% CI: 1.70–6.63; *p* < 0.001). These results reconsidered the value of TRG, enhancing the need for a more complex and global analysis of clinical and pathologic factors involving and underlining the undeniable importance of adequate lymph node resection for accurate staging and treatment of pts with GEJ cancer.

Among other studies that investigated the predictive value of molecular markers expression for treatment response and survival, great interest has been garnered by Phase II randomized trial FLOT4-AIO [43] with a significantly higher proportion of pts achieving pCR (according to Becker’s classification, 16% versus 6% in the group treated with docetaxel-based versus epirubicin-based triplet, respectively).

In conclusion, pathologic tumor response has been shown as a controversial but promising survival marker in GC. Larger and more standardized trials are needed in order to improve the reproducibility of the different scoring systems and to assess the prognostic value of this parameter.

### 6.3. Real Life Population: Treatment for Elderly Patients

GC is a disease of the elderly. In fact, according to the Surveillance, Epidemiology, and End Results (SEER) database [64], the median age at diagnosis is 68 years old—25.7% of the total cases develops in the age range of 65–75 years old and 34.5% of pts are more than 75 years of age.

Elderly pts with GC are often not included in clinical trials and international guidelines are mainly based on the results of treatment options in younger pts. Moreover, they tend to be undertreated (less aggressive surgery because of the risk of postoperative complications and less intense chemotherapy because of co morbidities), although age has not been identified as a prognostic factor in the outcome of metastatic or advanced GC. In this case, it is known that systemic chemotherapy improves OS and quality of life (QoL) of pts, compared to the best supportive care alone.

A pooled analysis of three clinical trials involving pts with metastatic or locally advanced GEJ cancer suggests that ≥70 years population treated with chemotherapy enjoys the same benefits in term of symptomatic response, tumor regression and survival compared to the younger, without relevant grade 3–4 toxicities [65]. Based on these results, the most important aspects to define in the older population are if the benefits are superior to the risks of treatment and how to select the most appropriate regimen.

MAGIC trial [7] was the first study to establish the superiority of perioperative ECF/X in terms of survival in resectable GC pts. This trial involved 20.4% of elderly pts (70 years of age) and did not show any differences for this subgroup in term of PFS or OS.

The FNCLCC/FFCD ACCORD trial [8], which involved 224 pts between 18 and 75 years of age with adenocarcinoma of the stomach, GEJ, or lower esophagus demonstrated a significant improvement in DFS and OS with perioperative treatment with CF doublet, followed by surgery, compared to surgery alone.

Recently, the German Phase III Trial, FLOT-4 Study [10,11], showed an important improvement in median PFS (30 versus 18 months, HR: 0.75; 95% IC: 0.62–0.91, *p* = 0.004) and median OS (50 versus 35 months, HR: 0.77; 95% IC: 0.63–0.94, *p*: 0.012), including 24% elderly pts. Moreover, the FLOT regimen has been already explored in the elderly population in the Phase II trial FLOT65+, in which 43 pts with advanced or metastatic GC were randomized to FLOT or FLO [66]. The primary end point of the study was to evaluate the tolerability and feasibility of the triplet, exploring the difference in terms of toxicity, severe adverse events, treatment discontinuation, and change in QoL and global health status, compared to the baseline. The results showed that the FLOT regimen was associated with an improvement of median PFS in the group with locally advanced disease (24.2 versus 10.3 months, *p*: 0.019) and in the under-70 years pts, whereas similar results were obtained in both populations in terms of response rate. As expected, pts in FLOT arm had a high rate of grade 3 or 4 neutropenia, leukopenia, nausea, and diarrhea (FLOT, 81.9%; FLO, 38.6%; *p*: 0.001). Nevertheless, no differences in treatment discontinuation were detected, except in the pts group of over-70 years (FLOT, 20.6%; FLO, 7.5%), wherein the addition of docetaxel seemed to have more toxicity that benefits, even if there was no worsening in QoL [67].

In conclusion, according to the limited published data, age is not considered an exclusion criterion to treat elderly pts with GC per se. Clinicians should focus on defining “fit or frail elderly”, according to global health and social status, cognitive function, co-morbidities and geriatric syndrome, globally known as multidisciplinary diagnostic assessment [68], with the aim to choose the best treatment for every patient.

### 6.4. Future Perspectives: Perioperative Treatment in The Oligometastatic Disease

Based on the “model” of perioperative approach for resectable GC, the role of multimodality treatment is also investigated in the setting of metastatic disease today. In fact, the integration of chemotherapy and surgery with a curative intent has been evaluated over the years in several retrospective experiences or subgroup analyses of clinical trials, showing a potential benefit, especially in the case of oligometastatic disease [69,70].

Recently, the AIO-FLOT 3 Phase II study prospectively stratified pts with untreated GC or GEJ cancer into three groups (operable (M0) pts, limited metastatic, or extensive metastatic pts); these pts received perioperative FLOT (four preoperative and four postoperative cycles) [71]. Limited metastatic disease was defined as distant intra-abdominal lymphnode metastases only or/and a maximum of one organ involved, normal serum alkaline phosphatase, <5 liver lesions, no visible carcinomatosis (peritoneum or pleura), and performance status according to the Eastern Cooperative Oncology Group (ECOG) ≤1. The trial showed that pts with limited metastatic disease who received neoadjuvant chemotherapy and proceeded to surgery (15% of the entire study population, 60% of pts with limited disease) had better survival than the others (median OS 31.3 months (95% CI: 18.9–NR) for pts who proceeded to surgery and 15.9 months (95% CI, 7.1–22.9) in pts who did not receive surgery). Based on these results, the Phase III RENAISSANCE trial (NCT02578368; EudraCT: 2014-002665-30), which is ongoing, aims to evaluate the effects of perioperative chemotherapy with FLOT in combination with curative gastrectomy/esophagectomy and resection of metastatic lesions or local ablation procedure in this setting [72].

## 7. Conclusions

Multimodality approach represents the standard of care for treatment of resectable GC pts today, especially in the case of proximal tumors. A multidisciplinary evaluation at medical centers with high expertise in the management of GC is mandatory at diagnosis, in order to evaluate patient and tumor characteristics, such as performance status, comorbidities, choice of patient, tumor site, staging, and pathological classifications; as well as the potential risks and benefits of each therapeutic approach. Based on these, pts with clinical T1, N0 tumors should proceed directly to surgery, with adjuvant therapy administration, if the pathological stage requires it; whereas pts with T3-4 or N+ tumors should be treated with a perioperative strategy with triplet-based chemotherapy. The choice of appropriate treatment in case of T2 is still under debate, although the most recent findings suggest the use of a perioperative approach in this case. Moreover, despite the lack of validated indications about the correct approach to use in pts with GC according to histotype, chemotherapy seems to be effective in case of diffuse GC with signet ring cells, and their exclusion from neoadjuvant chemotherapy on the basis of histology is not justified. Further research on predictive and prognostic factors, such as the role of early 18-FDG-PET assessment, definition of EBV, MMR and microsatellite status at diagnosis, and role of histology or pathological tumor response could help in the selection of pts for surgery or perioperative approach.

## Figures and Tables

**Table 1:** Landmark chemotherapy trials in perioperative setting.

Trial	Phase	Setting	Tumor Location	Regimens	Patients (*n*)	pCR	R0	mOS	mDFS
MAGIC [7]	III	Perioperative	Stomach: 74% GEJ: 11% Lower ES: 15%	ECF Surgery	250 253	Not reported	Not reported	5-y OS: 36% vs. 23%	
FFCD-ACCORD [8]	III	Perioperative	Stomach: 25% GEJ: 64% Lower ES: 11%	CF Surgery	113 111	Not reported	87% 74%	5-y OS: 38% vs. 24%	5-y DFS: 34% vs. 19%
OE05 [9]	III	Neoadjuvant	ES (including Siewert 1–2) 100%	ECX CF	446 451	7% 2%	66% 59%	26.1 months 23.4 months Not significant	14.4 months 11.6 months Not significant
FLOT4 [10,11]	III	Perioperative	GEJ: 56% Stomach: 44%	FLOT ECX/F	356 360	25% ^1^ 15%	Not available	50 months 35 months	30 months 18 months

^1^ Data for pCR of FLOT4 are obtained from ESMO 2017 presentation. Abbreviations: gastro-esophageal junction tumors (GEJ); esophagus (ES); pathological complete response (pCR); median overall survival (mOS); median disease-free survival (mDFS).

**Table 2:** Comparison between the main trials for chemotherapy and chemo-radiotherapy in a perioperative setting for gastric and gastroesophageal cancers.

**Trial**	MAGIC [7]	FFCD ACCORD [8]	EORTC [23]	CALGB [24]	CROSS [25,26]
**Year**	2006	2011	2010	2008	2012/Update 2015
**Approach**	CT triplet periop.	CT doublet periop.	CT doublet preop.	CRT	CRT
**Nation**	United Kingdom	France	Germany	US	The Netherlands
**Patients (*n*)**	503 pts	224 pts	144 pts	56 pts	368 pts
**Regimen**	ECFx3 → ECFx3	CFx4 → CFx4	PLFx4	CFx5 → RT 50.4 Gy	Weekly Carbo AUC2 + paclitaxel + RT 41.4 Gy
**Phase**	Phase III	Phase III	Phase III	Phase III	Phase III
**Most Common G3/4 Adverse Events ^1^**	Neutropenia: 24% Nausea: 6% Vomiting: 6% Anemia: 5% Diarrhea: 3%	Neutropenia: 20.2% Nausea/vomiting 9.2%. thrombocytopenia: 5.5%. Mucosite: 3.7%. Diarrhea: 1.8%.	Nausea: 5.5%. Vomiting: 5.5%. Renal toxicity: 2.8%. Cardiac toxicity: 1.4%	Neutropenia: 34%. Anemia: 15%. Infection: 30%. Thrombocytopenia: 11%. Nausea: 11%.	Anorexia: 5%. Fatigue: 3%. Diarrhea: 1%. Neutropenia: 2%. Nausea: 1%. Vomiting: 1%.
**Postoperative Mortality**	5.6% (vs. 5.9% in surgery alone arm)	25.7% (vs. 19.1% in surgery alone arm)	4.2% (vs. 1.4% in surgery alone arm)	0% (vs. 4,2% in surgery alone arm)	2% (vs. 3% in surgery arm)
**Results**	HR = 0.75 (95% CI: 0.60–0.93)	HR = 0.69 (95% CI: 0.50–0.96)	HR = 0.84 (95% CI: 0.52–1.35)	HR = 0.45 (95% CI: 0.20–1.01)	HR = 0.68 (95% CI: 0.53–0.88)

^1^ In case of perioperative chemotherapy, the adverse events (AE) refer exclusively to the preoperative phase.

**Table 3:** Landmark and ongoing trials of biological and immunological agents in perioperative treatment of gastric adenocarcinoma.

Her-2 Target Therapy								
Trial	Phase	Tumor Location	Regimens	Patients (*n*)	pCR	R0	mOS	mDFS
NEOHX [33]	II	Stomach: 21 pts Junction: 15 pts	Xelox ± trastuzumab	36	8.3%	-	NR	2-y: 60% NR
HER-FLOT [34]	II	Stomach: 18 pts Junction: 40 pts	FLOT ± trastuzumab	58	22.2%	93.3%	-	-
PETRARCA	II/III	ongoing	FLOT ± trastuzumab pertuzumab					
INNOVATION	II	ongoing	Cisplatin Fluorouracil ±trastuzumab pertuzumab					
**VEGF Target Therapy**								
ST03 [35]	III	Stomach: 36%	ECX/F ECX + bevacizumab	533 530	5% 3%	64% 61%	50.3% 48.1%	
RAMSES	II/III	ongoing	FLOT ± ramuciruab					
**Immune Checkpoint Inhibitors**								
KEYNOTE-585	III	ongoing	CF ± pembrolizumab FLOT ± pembrolizumab					
ICONIC	I/II	ongoing	FLOT + avelumab					
